# A New Computer-Based Cognitive Measure for Early Detection of Dementia Risk (Japan Cognitive Function Test): Validation Study

**DOI:** 10.2196/59015

**Published:** 2025-02-14

**Authors:** Hiroyuki Shimada, Takehiko Doi, Kota Tsutsumimoto, Keitaro Makino, Kenji Harada, Kouki Tomida, Masanori Morikawa, Hyuma Makizako

**Affiliations:** 1 Department of Preventive Gerontology, Centre for Gerontology and Social Science National Center for Geriatrics and Gerontology Ōbu Japan; 2 Department of Physical Therapy, School of Health Sciences, Faculty of Medicine Kagoshima University Kagoshima Japan

**Keywords:** cognition, neurocognitive test, dementia, Alzheimer disease, aged, MMSE, cognitive impairment, Mini-Mental State Examination, monitoring, eHealth

## Abstract

**Background:**

The emergence of disease-modifying treatment options for Alzheimer disease is creating a paradigm shift in strategies to identify patients with mild symptoms in primary care settings. Systematic reviews on digital cognitive tests reported that most showed diagnostic performance comparable with that of paper-and-pencil tests for mild cognitive impairment and dementia. However, most studies have small sample sizes, with fewer than 100 individuals, and are based on case-control or cross-sectional designs.

**Objective:**

This study aimed to examine the predictive validity of the Japanese Cognitive Function Test (J-Cog), a new computerized cognitive battery test, for dementia development.

**Methods:**

We randomly assigned 2520 older adults (average age 72.7, SD 6.7 years) to derivation and validation groups to determine and validate cutoff points for the onset of dementia. The Mini-Mental State Examination (MMSE) was used for comparison purposes. The J-Cog consists of 12 tasks that assess orientation, designation, attention and calculation, mental rotation, verbal fluency, sentence completion, working memory, logical reasoning, attention, common knowledge, word memory recall, and episodic memory recall. The onset of dementia was monitored for 60 months. In the derivation group, receiver operating characteristic curves were plotted to determine the MMSE and J-Cog cutoff points that best discriminated between the groups with and without dementia. In the validation group, Cox proportional regression models were developed to predict the associations of the group classified using the cutoff points of the J-Cog or MMSE with dementia incidence. Harrell C-statistic was estimated to summarize how well a predicted risk score described an observed sequence of events. The Akaike information criterion was calculated for relative goodness of fit, where lower absolute values indicate a better model fit.

**Results:**

Significant hazard ratios (HRs) for dementia incidence were found using the MMSE cutoff between 23 and 24 point (HR 1.93, 95% CI 1.13-3.27) and the J-Cog cutoff between 43 and 44 points (HR 2.42, 95% CI 1.50-3.93). In the total validation group, the C-statistic was above 0.8 for all cutoff points. Akaike information criterion with MMSE cutoff between 23 and 24 points as a reference showed a poor fit for MMSE cutoff between 28 and 29 points, and a good fit for the J-Cog cutoff between 43 and 44 points.

**Conclusions:**

The J-Cog has higher accuracy in predicting the development of dementia than the MMSE and has advantages for use in the community as a test of cognitive function, which can be administered by nonprofessionals.

## Introduction

Dementia is a syndrome associated with neurodegenerative diseases, characterized by a general decline in cognitive abilities, which affects daily activities. It ultimately has a significant impact on patients and their careers and social relationships. The most common symptoms of dementia include problems with memory, thinking, behavior, emotions, language, and reduced motivation [1]. The population of Japan is aging more rapidly than that of any other country. As of 2020, the number of Japanese people older than 65 years of age has reached a record high of 36 million, or 29% of the total population of 125 million. The number of people with dementia in Japan is projected to reach 7 million by 2025. To address this issue, the Basic Act for Dementia to Promote an Inclusive Society was approved on June 14, 2023, and took effect on January 1, 2024. This law requires national and local governments to ensure the social participation of people with dementia, develop a consultation system, and promote research for the development of a symbiotic society so that people with dementia can live with peace of mind and protect their human rights [2].

The Basic Act for Dementia to Promote an Inclusive Society includes measures to promote early detection, diagnosis, and response to dementia regarding prevention strategies. Early identification through screening would also allow patients and their families to receive care at an earlier stage in the disease process, potentially facilitating discussions regarding decision-making (eg, health care, financial, and legal), while the patient still has decision-making capacity [3]. Several progressive local governments have already initiated screening programs for the early detection of dementia risk. However, the cognitive function tests used as screening methods are Mini-Mental State Examination (MMSE) or similar conventional paper-and-pencil tests. The delay in digitizing cognitive tests might limit the number of older adults who are eligible for primary screening. A meta-analysis reported a high rate of undetected dementia, particularly in populations with low socioeconomic status [4]. This is likely due to the lack of widespread implementation of appropriate cognitive screening tests. Furthermore, the meta-analysis results suggested that studies that used the *Diagnostic and Statistical Manual of Mental Disorders, Third and* *Fourth Editions* criteria to identify dementia had a higher rate of underdetection than those that used the MMSE [4].

Lecanemab (Leqembi), a treatment agent for early Alzheimer disease (AD), can significantly alter the disease course for patients in the early stages [5]. Among participants with early symptomatic AD and amyloid and tau pathology, donanemab significantly slowed clinical progression in those with low or medium tau and in the combined low or medium and high tau pathology population [6]. The World Health Organization guidelines on risk reduction for cognitive decline and dementia provide evidence-based recommendations regarding lifestyle behaviors and interventions to delay or prevent cognitive decline and dementia [7]. These preventive paradigms create the need to identify patients with mild symptoms, typically in primary care settings, and refer them to AD specialists for formal diagnosis and to determine treatment or prevention eligibility.

Primary screening of cognitive function in the community is required to facilitate early hospital visits for early diagnosis of AD. The involvement of psychologists and other professionals in administering specialized neuropsychological assessments in the community is limited, and a screening test for cognitive function that can be administered by nonprofessionals would improve primary screening. To address these issues, cognitive testing as a digital biomarker that is reliable and self-administered and can detect cognitive dysfunction at an early stage is required. A review and recommendation on routine screening for cognitive impairment in adults aged 65 years and older by the US Preventive Services Task Force asks that clinicians remain alert to early signs of cognitive impairment for individual evaluation [3].

A systematic review on digital cognitive tests reported that most of these tests showed diagnostic performance comparable with that of paper-and-pencil tests for mild cognitive impairment (MCI) and dementia [8]. Furthermore, digital biomarkers related to memory and executive function might be more sensitive than those related to other cognitive domains [9]. These findings suggest that digital cognitive battery tests are promising sensitive clinical tools for detecting MCI and early dementia. However, most studies have small sample sizes, with fewer than 100 individuals, and are based on case-control or cross-sectional designs [9]. Further studies may require the use of digital cognitive biomarkers in long-term follow-up to monitor the conversion from normal cognition to dementia.

We developed a digital cognitive test, the Japanese Cognitive Function Test (J-Cog), which assesses orientation, verbal ability, attention, visuospatial performance, reasoning, and memory, to identify older adults with cognitive impairment in primary settings. The purpose of this study was to evaluate the effectiveness of the J-Cog in predicting the development of dementia and compare the accuracy of the J-Cog with that of the MMSE [10], one of the most widely used tests of cognitive function. If the detective validity of the J-Cog is confirmed, it can be widely used in the community as a cognitive screening tool.

## Methods

### Participants

In total, 4167 community-dwelling older adults who were recruited from Takahama City, Japan, for the National Center for Geriatric and Gerontology-Study of Geriatric Syndromes (NCGG-SGS) [11], which is a Japanese national cohort study, were enrolled in this study. The inclusion criteria were residence in Takahama and age ≥60 years at the time of the examination (September 2015 to February 2017). The exclusion criteria were (1) availing the Japanese certified public long-term care insurance system, which requires support or care (n=76); (2) not availing the certified public long-term care insurance system but having the inability to perform basic activities of daily living (such as eating, dressing, bathing, climbing stairs) and maintain functional mobility, personal hygiene, grooming, and toilet hygiene (n=9); (3) history of dementia (n=7) and the following conditions or symptoms suspected of causing dementia: Parkinson disease (n=16), stroke (n=219), or depression (n=97), and a score of <21 on the MMSE [12] (n=67); and (4) missing data for cognitive assessments and other measurements (n=1156). Of the initial 4167 participants, 1647 were excluded, and data from 2520 older adults were analyzed (1017 men and 1503 women). The mean age was 72.7 (SD 6.7; range 60-96) years. Analyses with dementia incidence as an outcome were conducted on 2292 participants who were available for follow-up.

### Measurements of Cognitive Function

#### Overview

Cognitive tests included the J-Cog and MMSE, administered once on the same day by well-trained assessors. The MMSE is commonly used to assess cognitive problems and is often a component of dementia diagnosis (range 0-30, higher scores indicating better functioning) [10].

#### Japanese Cognitive Function Test

##### Overview

The J-Cog was developed by neuropsychological researchers at the National Center for Geriatrics and Gerontology as a cognitive test for measuring the domains of cognitive impairment that are generally recognized in MCI. The initial version comprised 15 tasks, which were evaluated in terms of their simplicity, importance, and feasibility. Based on these assessments, the final version, comprising 12 tasks, was developed.

The J-Cog was presented on an iPad (Apple Inc) with a 9.7-inch touch display. The task instructions and questions were presented on the display with a letter size of at least 1.0 cm^2^. This battery consists of 12 tasks—orientation, designation, attention and calculation, mental rotation, verbal fluency, sentence completion, working memory, logical reasoning, attention, common knowledge, word memory recall, and episodic memory recall [13]—that assess cognitive functions that tend to decline with age. The participants were given 20-30 minutes to complete the battery (described further in this study). Higher J-Cog scores indicate better cognitive ability (range 0-72). An operator supported each participant in setting up the iPad, understanding the task protocols, and recording their data. The participants only needed to touch the display to complete the tasks using a digital pen. Because the J-Cog is a mixture of examinations in which the display of the screen is made invisible to the participant and examinations in which the display of the screen is shown to the participant, the examinations were conducted by well-trained operators.

##### Task 1: Orientation

The participants indicated the month, day, and weekday of the test date. The participants also indicated the prefecture and city of their current location. All 5 questions in each task were shown, and we calculated the total number of correct answers (0-5 points).

##### Task 2: Designation

The participants looked at pictures of eyeglasses, keys, wallets, watches, and cell phones and responded with the name of the object. One point was awarded for each item (0-5).

After naming the objects, they had 15 seconds to memorize the 5 objects they named. The participants then chose 1 of 5 items and answered where it would be stored, that is, under the television, on the dresser, on the shoebox, under the table, or in the dresser. These encoding tasks were scored during memory recall.

##### Task 3: Attention and Calculation

The participants responded to additions and subtractions of up to 2 digits by rote calculations. There were 6 questions, each presented only once (0-6 points). If there was obvious noise, the questions were presented again.

##### Task 4: Mental Rotation

The participants saw a completed figure with 3×3 and 4×4 squares showing kana (Japanese alphabet) and completed an incomplete figure with rotated kana missing (0-5 points). First, the participants were asked to answer example questions. If the answer to the example question was incorrect, the correct answer was taught, and the test was conducted after the participants understood the rules. The time limit was 20 seconds for the 3×3 task and 30 seconds for the 4×4 task.

##### Task 5: Verbal Fluency

The participants were asked to say as many words as possible to fit the category (eg, for vegetables, answer carrots, onions, and lettuce). They had 15 seconds to respond to as many categories as possible (0-20 points).

##### Task 6: Sentence Completion

The participants responded to the words that applied to the blanks in the sentence from 5 choices (0-2 points). They read a sentence with a missing word and selected the word that fits the blank based on the context of the sentence; 2 types of sentences were presented, each containing 5 choices.

The participants were asked to read the question silently and provide the correct answer. If they did not know the answer, they were asked to select “don’t know.” After each question, the participants were asked to press the next button to complete their full response.

##### Task 7: Working Memory

Participants were shown images of blue dots. After a while, some of the dots changed to white, then returned to blue. They were then asked to indicate which dots had changed to white. In addition, 2 questions were asked: one with 3×3 dots, and the other with 4×4 dots (0-7 points).

##### Task 8: Logical Reasoning

The participants responded to a logical reasoning text task and 4 fill-in-the-blank questions (0-4 points). Participants were asked to read the instructional text and question silently and select the correct response according to the instructions.

##### Task 9: Attention

The participants were shown 2 similar pictures and had 30 seconds to find the 1 shape present in only 1 of the pictures (0-1 points).

##### Task 10: Common Knowledge

The participants looked at pictures of national flags and were asked to choose the corresponding country from 8 options. There were 8 questions (0-8 points).

##### Task 11: Word Memory Recall

The participants responded to the 5 objects memorized in task 2 (0-5 points).

##### Task 12: Episodic Memory Recall

The participants responded with the object and location of the object that they stored away in task 2. If the participant were unable to answer, they listened to the options and selected the correct answer (0-4 points).

### Measurement of Incidence of Dementia

Participants who did not have dementia at baseline and were diagnosed with dementia during the 60-month, follow-up period were considered to have new-onset dementia. Data were collected from the Japanese National Health Insurance System and the Japanese long-term care insurance system. In Japan, all adults aged ≥65 years have public health insurance, which includes one of the following: health insurance for employed individuals (Employees’ Health Insurance), national health insurance for unemployed and self-employed individuals aged <75 years (Japanese National Health Insurance), or health care for individuals aged ≥75 years (Later-Stage Medical Care). In this study, participants were followed up monthly for new-onset dementia (AD, vascular dementia, frontotemporal dementia, or other dementia subtypes), as recorded by the Japanese National Health Insurance System and Later-Stage Medical Care. Participants were considered to have dementia based on a diagnosis made by a physician according to the *International Classification of Diseases, Tenth Revision*. We identified cases of dementia from the NCGG-SGS data according to the corresponding *International Classification of Diseases, Tenth Revision* codes (Multimedia Appendix 1). The diagnosis of dementia from the UK Biobank inpatient data, which have criteria similar to those of the NCGG-SGS (Multimedia Appendix 1), was previously validated and showed a positive predictive value of 87.3% for any dementia compared with the clinical expert adjudication of full-text medical records [14].

The long-term care insurance system is a mandatory form of social insurance that supports the daily lives of older adults with disabilities [15,16]. In Japan, all individuals aged ≥65 years are eligible for institutional or community-based services depending on their disability level, and those aged ≥40 years with disabilities related to aging, such as cerebrovascular disease, are also eligible to access benefits. The disability certification process consists of two parts: (1) the degree of disability based on a questionnaire developed by the Ministry of Health, Labor, and Welfare of Japan, and (2) a physician’s written opinion prepared by the attending physician [17]. We identified dementia based on the level of independence in the daily living of older adults with dementia in the disability certification process. The level of independence was classified into 5 ranks, from mild to severe (I, II, III, IV, and M); individuals with moderate dementia-related behavioral disturbances and cognitive impairment with slight dependence were classified as rank II. We defined dementia as symptoms of rank II or higher, as derived from a primary care physician’s written opinion. These criteria were the same as those used in previous studies in Japan [18-21].

### Analysis of Potential Confounding Factors

Dementia results from a combination of factors that occur over time, including demographics and chronic conditions. All multivariate-adjusted models reported included the following covariates unless otherwise noted: age at enrollment, sex, educational level, drinking, smoking, presence of chronic conditions, BMI, and regular exercise. The self-reported chronic conditions included in the model were hypertension, diabetes, cancer, and osteoarthritis [22,23].

### Ethical Considerations

This study was approved by the ethics committee of the Institutional Review Board of the National Center for Geriatrics and Gerontology (approval 770). All the participants were informed of the study and provided their written consent before their participation in this study, agreeing to the anonymous use of their data for publication. Data were deidentified before analysis and stored securely in a password-protected file on a password-protected computer. No compensation was provided to the study participants.

### Statistical Analysis

First, those who participated at baseline and could follow the development of dementia during the follow-up period were randomly divided into the derivation and validation groups (1:1). Data homogeneity analysis regarding the control variables and J-Cog scores was tested using independent sample 2-tailed *t* tests or chi-square tests for contingency tests, depending on the variable structure. Incident dementia diagnoses per 1000 person-years were calculated for all participants and for participants classified by the cutoff point. Chi-square tests were used to compare the groups for incident dementia.

In the derivation group, receiver operating characteristic curves were plotted to determine the MMSE and J-Cog cutoff points that best discriminated between groups with and without dementia (the cutoff points are denoted with the 2 points on either side of the cutoff separated by a “/”). The area under the receiver operating characteristic curve was calculated, and the cutoff points for maximizing the sensitivity and specificity of each test were determined using the Youden index [24]. Based on these cutoff points, participants were categorized as having either high or low cognitive ability. We compared the accuracy of predicting the development of dementia by classifying participants using the MMSE cutoff point 23/24, which is used to determine the risk of dementia [10].

In the validation group, 3 Cox proportional regression models were developed to predict the associations of the group (lower or higher cognitive ability) classified using the J-Cog or MMSE with dementia incidence. Specifically, the first and second models used MMSE score cutoffs of 23/24 and 28/29, respectively, and the third model used a J-Cog score cutoff of 43/44. All the models were adjusted for the potential confounding factors mentioned above. The adjusted hazard ratio (HR) and 95% CIs were obtained from the respective models. Harrell C-statistic was estimated to summarize how well a predicted risk score described an observed sequence of events. Generally, a C-statistic value greater than 0.7 indicates a good match [25,26]. The Akaike information criterion (AIC) was calculated for relative goodness of fit, where lower absolute values indicate a better model fit. Differences in Akaike information criterion (ΔAIC) of 3 or more were considered meaningful [27]. Harrell C-statistic and AIC were calculated using the *dynpred* (version 0.1.2) and *survival* (version 3.5.7) R package [28,29]. As sensitivity analyses, Cox proportional regression models were used, stratified by age group (65-74 years vs 75 years or more) and educational history (less than 9 years vs 10 years or more). These are variables that have a robust relationship with cognitive decline and dementia [30,31], and we felt that the stratified analyses would show the benefits of population-specific cognitive testing.

All statistical tests were considered significant at *P*<.05, and statistical analyses were performed using IBM SPSS Statistics (version 24.0) and R version 4.3.2 (R Core Team).

## Results

**Figure 1:**
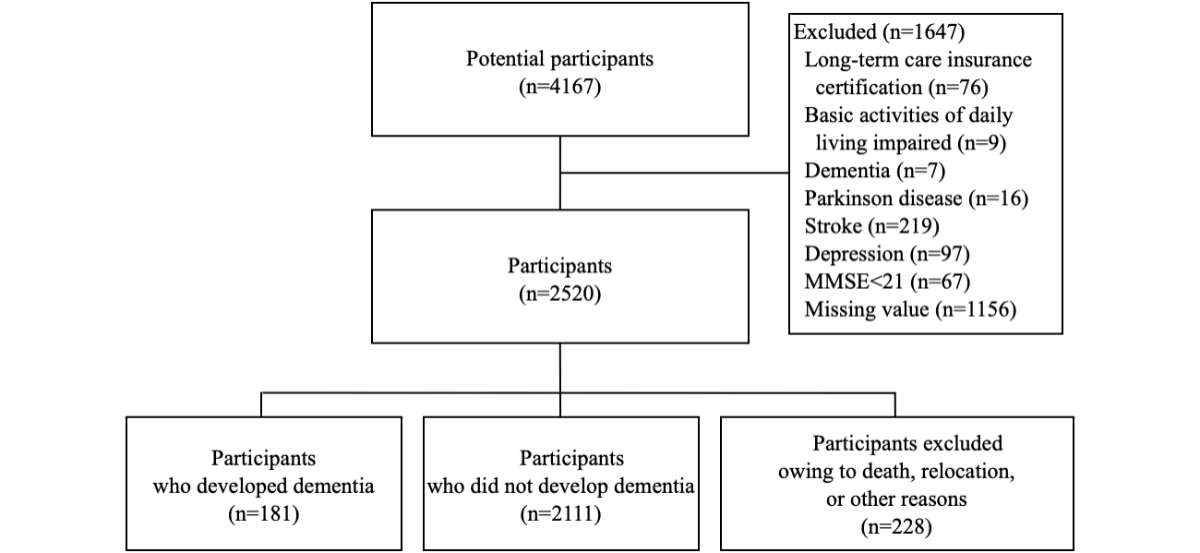
Flow chart of participant enrollment. MMSE: Mini-Mental State Examination.

Of the 2520 participants, 181 (7.2%) were diagnosed with dementia, 29 (1.2%) moved away from Takahama City, 138 (5.5%) died during the follow-up period, and 61 (2.4%) terminated the study for other reasons (Figure 1). Incident dementia diagnosis per 1000 person-years (95% CI) for total participants was 15.7 (13.6-18.1).

We examined whether the possible confounders of dementia incidence and cognitive tests differed at baseline between the derivation and validation groups (Table 1). No significant differences were observed in any of the measurements between the 2 groups (all *P*>.05). Table 2 presents the possible confounding factors for dementia incidence and cognitive test results for participants grouped according to the presence or absence of dementia. In the derivation group, significant differences were found for age (*P*<.001), educational level (*P*<.001), drinking (*P*=.02), diabetes (*P*=.002), cancer (*P*=.04), MMSE (*P*<.001), all subscores in the J-Cog (all *P*<.05), and total J-Cog score (*P*<.001) between participants with and without dementia (Table 2). In the validation group, significant differences were found for age (*P*<.001), sex (*P*=.02), education level (*P*<.001), hypertension (*P*=.05), osteoarthritis (*P*<.001), MMSE, and all subscores in the J-Cog (all *P*<.05) except for orientation and designation, and total J-Cog score (*P*<.001) between the participants with and without dementia incidence (Table 2).

The Youden index was used to determine the cutoff points for future dementia incidence during a 60-month period, which were 28/29 and 43/44 points on the MMSE and J-Cog, respectively. Incident dementia diagnoses per 1000 person-years with 95% CI for the low and high cognitive ability groups were 21.0 (17.9-24.6) and 7.1 (5.1-10.0) according to the MMSE 28/29 cutoff, 35.0 (29.5-41.4) and 6.0 (4.5-8.0) according to the J-Cog 43/44 cutoff, and 42.5 (31.1-58.0) and 13.2 (11.2-15.5) according to the MMSE 23/24 cutoff. The low cognitive ability groups, which were divided according to the 3 cutoff points, included significantly more participants with incident dementia than those with high cognitive ability (*P*<.001).

**Figure 2:**
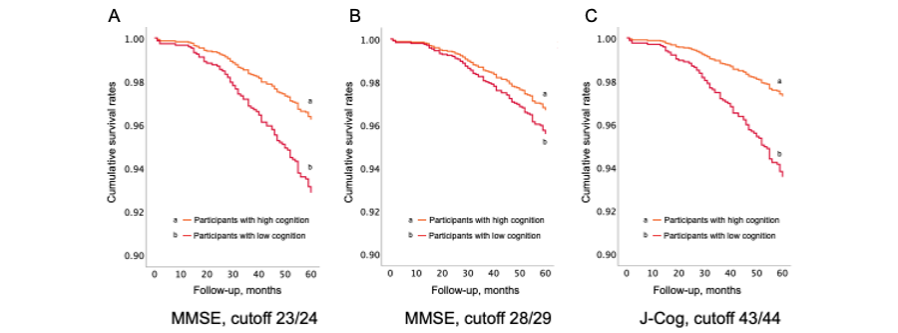
Cox survival estimates for dementia incidence according to cognitive ability: (A) hazard estimates of dementia incidence with MMSE cutoff point 23/24; (B) hazard estimates of dementia incidence with MMSE cutoff point 28/29; and (C) hazard estimates of dementia incidence with J-Cog cutoff point 43/44. The cutoff points are denoted by the 2 points on either side of the cutoff separated by a “/”. J-Cog: Japanese Cognitive Function Test; MMSE: Mini-Mental State Examination.

The predictive performances of all 3 risk cutoff points for dementia are presented in Table 3. In the total validation group participants, significant HRs for dementia incidence were found using the MMSE 23/24 and J-Cog 43/44 cutoffs (MMSE 23/24: HR 1.93, 95% CI 1.13-3.27; J-Cog 434/44: HR 2.42, 95% CI 1.50-3.93; Figure 2). The C-statistic was above 0.7 for all cutoff points. The ΔAIC with the MMSE 23/24 cutoff as a reference indicated that the MMSE 28/29 cutoff had poor discrimination, and the J-Cog 43/44 cutoff had good discrimination.

A subgroup analysis of participants aged 65-74 years showed no significant HRs (HR 1.31, 95% CI 0.53-3.22) at the MMSE 28/29 cutoff and a poor fit (ΔAIC=5.6). Participants aged 75 years and older showed a good fit at the J-Cog 43/44 cutoff (ΔAIC=–23.2), with significant HRs (HR 2.71, 95% CI 1.84-4.00) and good discrimination (C-statistics=0.714). In participants with less than 9 years of education, significant HRs for dementia incidence were found at the MMSE 23/24 and J-Cog 43/44 cutoff points (MMSE 23/24: HR 1.83, 95% CI 1.18-2.83; and J-Cog 43/44: HR 2.26, 95% CI 1.38-3.69). The ΔAIC using the MMSE 23/24 cutoff as a reference showed a poor fit at the MMSE 28/29 cutoff (ΔAIC=4.9) and a good fit at the J-Cog 43/44 cutoff (ΔAIC=–5.3). In participants with more than 10 years of education, significant HRs for dementia incidence were found at the MMSE 28/29 and J-Cog 43/44 cutoff points (MMSE 28/29: HR 1.84, 95% CI 1.06-3.22; and J-Cog 43/44: HR 3.53, 95% CI 2.10-5.93). The ΔAIC using the MMSE 23/24 cutoff as a reference showed good fit at the MMSE 28/29 (ΔAIC=–3.5) and J-Cog 43/44 cutoff points (ΔAIC=–22.7).

**Table 1:** Baseline characteristics of the participants.

Characteristic		Derivation group (n=1260)	Validation group (n=1260)	*P* value
**Age, years (mean, SD)**		72.7 (6.7)	72.7 (6.7)	.78
**Sex, female (n, %)**		756 (60)	747 (59.3)	.72
**Education, years (mean, SD)**		11.0 (2.3)	11.1 (2.4)	.44
**Drinking, no (n, %)**		863 (68.5)	865 (68.7)	.93
**Smoking, yes (n, %)**		121 (9.6)	111 (8.8)	.49
**Hypertension, yes (n, %)**		611 (48.5)	613 (48.7)	.94
**Diabetes, yes (n, %)**		182 (14.4)	171 (13.6)	.53
**Cancer, yes (n, %)**		152 (12.1)	161 (12.8)	.59
**Osteoarthritis, yes (n, %)**		235 (18.7)	236 (18.7)	.96
**BMI (mean, SD)**		23.4 (3.2)	23.5 (3.3)	.59
**Regular exercise, no (n, %)**		299 (23.7)	312 (24.8)	.55
**Mini-mental state examination, point (mean, SD)**		27.1 (2.4)	27.2 (2.4)	.40
**J-Cog^a^** **, point (mean, SD)**				
	Orientation	5.0 (0.3)	4.9 (0.3)	.35
	Designation	5.0 (0.2)	5.0 (0.2)	.27
	Attention and calculation	4.2 (1.6)	4.2 (1.6)	.68
	Mental rotation	3.8 (1.5)	3.8 (1.5)	.79
	Verbal fluency	2.7 (1.0)	2.7 (1.0)	.84
	Sentence completion	1.6 (0.5)	1.6 (0.5)	.38
	Working memory	6.7 (0.7)	6.7 (0.6)	.12
	Logical reasoning	3.5 (0.7)	3.5 (0.7)	.89
	Attention	0.7 (0.5)	0.7 (0.5)	.28
	Common knowledge	5.0 (1.7)	5.1 (1.8)	.20
	Word and episodic memory recall	6.9 (1.9)	7.0 (1.9)	.63
	Total	45.0 (6.0)	45.2 (6.0)	.35

^a^J-Cog: Japanese Cognitive Function Test.

**Table 2:** Baseline characteristics of study participants stratified by development of dementia.

Characteristic		Derivation group (n=1143)				Validation group (n=1149)		
		Participants who did not develop dementia (n=1051)	Participants who developed dementia (n=92)	*P* value	Participants who did not develop dementia (n=1060)		Participants who developed dementia (n=89)	*P* value
**Age (years), mean (SD)**		71.8 (6.3)	80.3 (4.7)	<.001	72.0 (6.3)		79.7 (5.4)	<.001
**Sex (female), n (%)**		643 (61.2)	54 (59)	.64	630 (59.4)		64 (72)	.02
**Education (years), mean (SD)**		11.1 (2.3)	10.2 (2.4)	<.001	11.2 (2.4)		10.2 (2.1)	<.001
**Drinking (no), n (%)**		706 (67.2)	73 (79)	.02	733 (69.2)		70 (79)	.06
**Smoking (yes), n (%)**		97 (9.2)	8 (9)	.87	974 (91.9)		82 (92)	.93
**Hypertension (yes), n (%)**		497 (47.3)	49 (53)	.27	503 (47.5)		52 (58)	.05
**Diabetes (yes), n (%)**		137 (13)	23 (25)	.002	141 (13.3)		11 (12)	.80
**Cancer (yes), n (%)**		119 (11.3)	17 (19)	.04	133 (12.5)		9 (10)	.503
**Osteoarthritis (yes), n (%)**		186 (17.7)	23 (25)	.08	187 (17.6)		31 (35)	<.001
**BMI (kg/m** ^**2**^ **), mean (SD)**		23.4 (3.2)	23 (3.3)	.18	23.6 (3.3)		23 (3.5)	.12
**Regular exercise (no), n (%)**		245 (23.3)	25 (27)	.40	261 (24.6)		22 (25)	.98
**Mini-Mental State Examination (point), mean (SD)**		27.3 (2.3)	25.8 (2.5)	<.001	27.3 (2.3)		26.0 (2.7)	<.001
**J-Cog^a^** **(point), mean (SD)**								
	Orientation	5.0 (0.2)	4.8 (0.5)	.005	5.0 (0.3)		4.9 (0.4)	.15
	Designation	5.0 (0.1)	4.9 (0.3)	.04	5.0 (0.2)		4.9 (0.3)	.12
	Attention and calculation	4.3 (1.6)	3.6 (1.7)	<.001	4.3 (1.5)		3.6 (1.6)	<.001
	Mental rotation	3.9 (1.4)	2.8 (1.8)	<.001	3.9 (1.5)		3.0 (1.7)	<.001
	Verbal fluency	2.8 (1.0)	2.1 (0.9)	<.001	2.8 (0.9)		2.2 (0.9)	<.001
	Sentence completion	1.6 (0.5)	1.4 (0.6)	<.001	1.7 (0.5)		1.4 (0.6)	<.001
	Working memory	6.7 (0.6)	6.3 (0.9)	<.001	6.8 (0.6)		6.5 (0.6)	.001
	Logical reasoning	3.5 (0.7)	3.1 (1.0)	<.001	3.5 (0.7)		3.3 (0.8)	.007
	Attention	0.7 (0.5)	0.5 (0.5)	.003	0.7 (0.5)		0.5 (0.5)	<.001
	Common knowledge	5.1 (1.7)	4.1 (1.7)	<.001	5.2 (1.7)		4.2 (1.7)	<.001
	Word and episodic memory recall	7.1 (1.7)	5.1 (2.2)	<.001	7.1 (1.8)		5.4 (2.2)	<.001
	Total	45.7 (5.7)	39.0 (6.1)	<.001	45.8 (5.8)		39.8 (6.1)	<.001

^a^J-Cog: Japanese Cognitive Function Test.

**Table 3:** Hazard ratio for development of dementia in the low and high cognitive ability groups.

Group and cutoff points		HR^a^ (95% CI)	*P* value	C-statistic	AIC^b^	ΔAIC^c^
**Validation group**						
	MMSE^d^ 23/24	1.93 (1.13-3.27)	.02	0.82	1147.6	Reference
	MMSE 28/29	1.34 (0.80-2.24)	.26	0.82	1151.7	4.1^e^
	J-Cog^f^ 43/44	2.42 (1.50-3.93)	<.001	0.83	1139.2	–8.4^g^
**Participants aged 65-74 years**						
	MMSE 23/24	5.30 (1.61-17.38)	.006	0.83	306.1	Reference
	MMSE 28/29	1.31 (0.53-3.22)	.56	0.83	311.7	5.6^e^
	J-Cog 43/44	2.86 (1.13-7.22)	.03	0.84	307.2	1.1
**Participants aged 75 years and older**						
	MMSE 23/24	1.64 (1.11-2.43)	.01	0.69	2110.9	Reference
	MMSE 28/29	1.69 (1.10-2.62)	.02	0.69	2110.5	–0.4
	J-Cog 43/44	2.71 (1.84-4.00)	<.001	0.71	2087.7	–23.2^g^
**Participants with less than 9 years of education**						
	MMSE 23/24	1.83 (1.18-2.83)	.007	0.81	1348.5	Reference
	MMSE 28/29	1.43 (0.83-2.46)	.20	0.81	1353.4	4.9^e^
	J-Cog 43/44	2.26 (1.38-3.69)	.001	0.81	1343.1	–5.3^g^
**Participants with 10 or more years of education**						
	MMSE 23/24	1.50 (0.72-3.11)	.28	0.84	964	Reference
	MMSE 28/29	1.84 (1.06-3.22)	.03	0.85	960.5	–3.5^g^
	J-Cog 43/44	3.53 (2.10-5.93)	<.001	0.87	941.3	–22.7^g^

^a^HR: hazard ratio.

^b^AIC: Akaike information criterion.

^c^ΔAIC: differences in Akaike information criterion.

^d^MMSE: Mini-Mental State Examination.

^e^Poor fit compared with Mini-Mental State Examination cutoff point 23/24 (ie, differences in Akaike information criterion greater than 3).

^f^J-Cog: Japanese Cognitive Function Test.

^g^Good fit compared with Mini-Mental State Examination cutoff point 23/24 (ie, differences in Akaike information criterion greater than –3).

## Discussion

### Principal Findings

In this study, we developed and validated the J-Cog to establish a digital cognitive marker with a low economic and human burden to serve a large number of older adults. The J-Cog is a promising free tool for use in community centers and primary care clinics so that at-risk older adults can receive timely cognitive screening in the community. This study showed that the J-Cog has good criteria and predictive validity for identifying older adults who have developed dementia. Automated administration and scoring algorithms can reduce the workload of professionals and health care staff.

In this prospective study, dementia risk was significantly associated with the MMSE 23/24 and J-Cog 43/44 cutoff points, and a good fit at the J-Cog 43/44 cutoff point. The advantages of the MMSE include rapid administration and high acceptance as a diagnostic tool among health professionals and researchers [32]. Traditionally, a 23/24 cutoff has been used to select patients with suspected cognitive impairment or dementia [33]. However, sociocultural variables, such as age and education, among others, may affect individual scores [34,35]. A review to determine the accuracy of MMSE for the early detection of dementia reported no evidence supporting the substantial role of the MMSE as a stand-alone, single-administration test in the identification of participants who could develop dementia [36].

The optimal cutoff point for the development of dementia in this study was a high MMSE score of 28/29. The mean MMSE score was 27 (SD 2), and the median score was 28 (IQR 25-29). Only 211 (8.4%) of 2520 participants had MMSE scores below 23, which is traditionally considered a high-risk factor for dementia [10]. The reason for the higher cutoff point in this study may be the high distribution of MMSE scores.

The MMSE cutoff point of 23/24 showed better accuracy than 28/29 for participants in the total validation group, those aged 65-74 years, and those with less than 9 years of education. These results suggest that the MMSE 23/24 cutoff is a robust indicator of the development of dementia. However, the MMSE 28/29 cutoff point of showed a better fit than that of 23/24 for participants with more than 10 years of education. This result is in line with that of a previous study that reported the need for a higher MMSE cutoff for diagnosing dementia for patients with high educational levels [37].

The J-Cog showed a better fit for detecting the onset of dementia than the MMSE 23/24 cutoff points. The findings were similar in analyses in which participants were stratified by age and educational level, indicating the superiority of the J-Cog over MMSE in predicting incident dementia. In addition to predictive validity, the J-Cog has certain advantages over pen-and-paper cognitive function tests, such as the fact that (1) it does not require a professional to administer the test, (2) it can be administered free of charge, and (3) scoring and judgment are automated. These benefits are important for covering a large number of participants in a community setting for cognitive testing.

### Comparison With Previous Work

The emergence of disease-modifying treatment options for AD has led to a paradigm shift in treatment strategies. Amyloid-targeted therapies are ideally used in the early stages of the disease, at the stage of MCI, and no later than mild dementia, because they slow disease progression but cannot reverse decline [38]. This preventive paradigm creates the need to identify patients with mild symptoms, typically in primary care settings, and many digital biomarkers have been developed. Systematic reviews on digital cognitive tests reported that most showed diagnostic performance comparable with that of paper-and-pencil tests for MCI and dementia. However, most studies have small sample sizes of fewer than 100 individuals and are based on case-control or cross-sectional designs [8,9]. In this study, a large population was followed for 60 months after implementation of the J-Cog to determine the predictive validity of the development of dementia in community-dwelling older adults. A recent systematic review reported the evaluation of digital cognitive testing applications, with a particular focus on the older people [39]. The review included an analysis of 20 articles. The number of cognitive tasks included in the accepted digital cognitive tests ranged from 1 to 10, with only 2 (10%) tests including more than 8 tasks. The cognitive domains included executive functions, memory, visuospatial, attention, language, orientation, processing speed, learning, praxis or gnosis, abstractions, and calculations [39]. Many of these domains were included in the J-Cog. Previous studies have not found any validation of predictive validity with dementia onset as an outcome. This study provides the first evidence that digital cognitive tests are a tool that can predict the future onset of dementia.

### Limitations

Although the J-Cog has some advantages over pen-and-paper cognitive function tests, there are some limitations to interpreting the results of this study. First, the participants were not randomly recruited, which may have resulted in an underrepresentation of cognitive impairment, because the participants were sufficiently healthy to undergo health examinations. Second, data on dementia subtypes were not collected (eg, AD, vascular dementia, dementia with Lewy bodies, and frontotemporal dementia). Thus, the data do not allow inferences regarding the correlation between the J-Cog and dementia pathology. Third, we were unable to contact an informant (eg, a family member) to verify the medical records, lifestyle information, or asymptomatic aberrant behavior. In addition, this study only identified dementia using specific *International Classification of Diseases, Tenth Revision* codes and information from the long-term care insurance system, so it was not possible to identify the onset of dementia in people who had dementia due to other factors or who had no record. These may have led to an underestimation of early-onset dementia in particular. Finally, information about the participants’ medical conditions and comorbidities was collected through self-reports, and because we did not have access to medical records, we were unable to confirm these reports.

### Conclusions

In this study, the J-Cog, a computerized cognitive battery test, was shown to be valid for detecting dementia development. The J-Cog is a cognitive function test that can be widely used in the community, and its social implementation should be promoted in the future so that AD treatment can be implemented in a timely and appropriate manner. A future extension of this study would be to improve the usability of the J-Cog so that it could be administered remotely and to remove the need for an operator to improve efficiency compared with paper-and-pencil tasks.

## Appendix

Multimedia Appendix 1 ICD-10 codes used to identify dementia cases in the UK Biobank and National Center for Geriatric and Gerontology-Study of Geriatric Syndromes. ICD-10: International Statistical Classification of Diseases, Tenth Revision.

## Abbreviations

AD: Alzheimer disease
AIC: Akaike information criterion
ΔAIC: differences in Akaike information criterion
HR: hazard ratio
J-Cog: Japanese Cognitive Function Test
MCI: mild cognitive impairment
MMSE: Mini-Mental State Examination
NCGG-SGS: National Center for Geriatric and Gerontology-Study of Geriatric Syndromes

## References

[1] World Health Organization (2017). Global Action Plan on the Public Health Response to Dementia 2017 - 2025.

[2] Ministry of Justice The Basic Act on Dementia to Promote an Inclusive Society (outline). Japanese Law Translation Database System.

[3] Owens DK, Davidson KW, Krist AH, Barry MJ, Cabana M, Caughey AB, Doubeni CA, Epling JW, Kubik M, Landefeld CS, Mangione CM, Pbert L, Silverstein M, Simon MA, Tseng C, Wong JB, US Preventive Services Task Force (2020). Screening for cognitive impairment in older adults: US preventive services task force recommendation statement. JAMA.

[4] Lang L, Clifford A, Wei L, Zhang D, Leung D, Augustine G, Danat IM, Zhou W, Copeland JR, Anstey KJ, Chen R (2017). Prevalence and determinants of undetected dementia in the community: a systematic literature review and a meta-analysis. BMJ Open.

[5] van Dyck CH, Swanson CJ, Aisen P, Bateman RJ, Chen C, Gee M, Kanekiyo M, Li D, Reyderman L, Cohen S, Froelich L, Katayama S, Sabbagh M, Vellas B, Watson D, Dhadda S, Irizarry M, Kramer LD, Iwatsubo T (2023). Lecanemab in early Alzheimer's disease. N Engl J Med.

[6] Sims JR, Zimmer JA, Evans CD, Lu M, Ardayfio P, Sparks J, Wessels AM, Shcherbinin S, Wang H, Monkul Nery ES, Collins EC, Solomon P, Salloway S, Apostolova LG, Hansson O, Ritchie C, Brooks DA, Mintun M, Skovronsky DM (2023). Donanemab in early symptomatic Alzheimer disease: The TRAILBLAZER-ALZ 2 randomized clinical trial. JAMA.

[7] WHO (2019). Risk Reduction of Cognitive Decline and Dementia: WHO Guidelines.

[8] Chan JYC, Yau STY, Kwok TCY, Tsoi KKF (2021). Diagnostic performance of digital cognitive tests for the identification of MCI and dementia: a systematic review. Ageing Res Rev.

[9] Ding Z, Lee TL, Chan AS (2022). Digital cognitive biomarker for mild cognitive impairments and dementia: a systematic review. J Clin Med.

[10] Folstein MF, Folstein SE, McHugh PR (1975). "Mini-mental state". A practical method for grading the cognitive state of patients for the clinician. J Psychiatr Res.

[11] Shimada H, Makizako H, Lee S, Doi T, Lee S, Tsutsumimoto K, Harada K, Hotta R, Bae S, Nakakubo S, Harada K, Suzuki T (2016). Impact of cognitive frailty on daily activities in older persons. J Nutr Health Aging.

[12] Shimada H, Tsutsumimoto K, Lee S, Doi T, Makizako H, Lee S, Harada K, Hotta R, Bae S, Nakakubo S, Uemura K, Park H, Suzuki T (2016). Driving continuity in cognitively impaired older drivers. Geriatr Gerontol Int.

[13] Lezak MD (2004). Neuropsychological Assessment.

[14] Wilkinson T, Schnier C, Bush K, Rannikmäe K, Henshall DE, Lerpiniere C, Allen NE, Flaig R, Russ TC, Bathgate D, Pal S, O'Brien JT, Sudlow CLM (2019). Identifying dementia outcomes in UK Biobank: a validation study of primary care, hospital admissions and mortality data. Eur J Epidemiol.

[15] Ikegami N (1997). Public long-term care insurance in Japan. JAMA.

[16] Tsutsui T, Muramatsu N (2005). Care-needs certification in the long-term care insurance system of Japan. J Am Geriatr Soc.

[17] Moriyama Y, Tamiya N, Kamimura A, Sandoval F, Luptak M (2014). Doctors' opinion papers in long-term care need certification in Japan: comparison between clinic and advanced treatment hospital settings. Public Policy and Administration Research.

[18] Ikeda A, Yamagishi K, Tanigawa T, Cui R, Yao M, Noda H, Umesawa M, Chei C, Yokota K, Shiina Y, Harada M, Murata K, Asada T, Shimamoto T, Iso H (2008). Cigarette smoking and risk of disabling dementia in a Japanese rural community: a nested case-control study. Cerebrovasc Dis.

[19] Yamamoto T, Kondo K, Hirai H, Nakade M, Aida J, Hirata Y (2012). Association between self-reported dental health status and onset of dementia: a 4-year prospective cohort study of older Japanese adults from the Aichi Gerontological Evaluation Study (AGES) project. Psychosom Med.

[20] Yamagishi K, Ikeda A, Chei CL, Noda H, Umesawa M, Cui R, Muraki I, Ohira T, Imano H, Sankai T, Okada T, Tanigawa T, Kitamura A, Kiyama M, Iso H (2017). Serum α-linolenic and other ω-3 fatty acids, and risk of disabling dementia: community-based nested case-control study. Clin Nutr.

[21] Ihira H, Sawada N, Inoue M, Yasuda N, Yamagishi K, Charvat H, Iwasaki M, Tsugane S (2022). Association between physical activity and risk of disabling dementia in Japan. JAMA Netw Open.

[22] Peters R, Booth A, Rockwood K, Peters J, D'Este C, Anstey KJ (2019). Combining modifiable risk factors and risk of dementia: a systematic review and meta-analysis. BMJ Open.

[23] Anstey KJ, Ee N, Eramudugolla R, Jagger C, Peters R (2019). A systematic review of meta-analyses that evaluate risk factors for dementia to evaluate the quantity, quality, and global representativeness of evidence. J Alzheimers Dis.

[24] Perkins NJ, Schisterman EF (2006). The inconsistency of "optimal" cutpoints obtained using two criteria based on the receiver operating characteristic curve. Am J Epidemiol.

[25] Longato E, Vettoretti M, Di Camillo B (2020). A practical perspective on the concordance index for the evaluation and selection of prognostic time-to-event models. J Biomed Inform.

[26] Sun W, Jiang YZ, Liu YR, Ma D, Shao ZM (2016). Nomograms to estimate long-term overall survival and breast cancer-specific survival of patients with luminal breast cancer. Oncotarget.

[27] Fayosse A, Nguyen DP, Dugravot A, Dumurgier J, Tabak AG, Kivimäki M, Sabia S, Singh-Manoux A (2020). Risk prediction models for dementia: role of age and cardiometabolic risk factors. BMC Med.

[28] Putter H (2011). dynpred: companion package to "Dynamic Prediction in Clinical Survival Analysis". R Project.

[29] Therneau TM, Lumley T, Elizabeth A, Cynthia C (2011). survival: survival analysis. R Project.

[30] Alzheimer's Association (2020). 2020 Alzheimer's disease facts and figures. Alzheimers Dement.

[31] Makizako H, Shimada H, Doi T, Tsutsumimoto K, Lee S, Hotta R, Nakakubo S, Harada K, Lee S, Bae S, Harada K, Suzuki T (2015). Cognitive functioning and walking speed in older adults as predictors of limitations in self-reported instrumental activity of daily living: prospective findings from the obu study of health promotion for the elderly. Int J Environ Res Public Health.

[32] Nieuwenhuis-Mark RE (2010). The death knoll for the MMSE: has it outlived its purpose?. J Geriatr Psychiatry Neurol.

[33] Tombaugh TN, McIntyre NJ (1992). The Mini-Mental State Examination: a comprehensive review. J Am Geriatr Soc.

[34] Brayne C, Calloway P (1990). The association of education and socioeconomic status with the Mini Mental State Examination and the clinical diagnosis of dementia in elderly people. Age Ageing.

[35] Bleecker ML, Bolla-Wilson K, Kawas C, Agnew J (1988). Age-specific norms for the Mini-Mental State Exam. Neurology.

[36] Arevalo-Rodriguez I, Smailagic N, Roqué-Figuls M, Ciapponi A, Sanchez-Perez E, Giannakou A, Pedraza OL, Bonfill Cosp X, Cullum S (2021). Mini-Mental State Examination (MMSE) for the early detection of dementia in people with mild cognitive impairment (MCI). Cochrane Database Syst Rev.

[37] Kochhann R, Varela JS, Lisboa CSDM, Chaves MLF (2010). The Mini Mental State Examination: review of cutoff points adjusted for schooling in a large Southern Brazilian sample. Dement Neuropsychol.

[38] Cummings J, Rabinovici GD, Atri A, Aisen P, Apostolova LG, Hendrix S, Sabbagh M, Selkoe D, Weiner M, Salloway S (2022). Aducanumab: appropriate use recommendations update. J Prev Alzheimers Dis.

[39] Cubillos C, Rienzo A (2023). Digital cognitive assessment tests for older adults: systematic literature review. JMIR Ment Health.

