# Supplementary material for: A New Computer-Based Cognitive Measure for Early Detection of Dementia Risk (Japan Cognitive Function Test): Validation Study
Source: J Med Internet Res. 2025 Feb 14;27:e59015. doi: 10.2196/59015 (PMC11888094; doi:10.2196/59015)
Supplement: Multimedia Appendix 1 [file jmir_v27i1e59015_app1.docx]

**Multimedia Appendix 1.** ICD-10 codes used to identify dementia cases in UK Biobank and National Center for Geriatric and Gerontology-Study of Geriatric Syndromes

| **ICD-10** | **Description** | **Subtype** | **UKB*** | **NCGG–SGS** | | |
| --- | --- | --- | --- | --- | --- | --- |
|  |  |  |  | **JNHI** | **LSMC** | |
| F00 | Dementia in Alzheimer's disease | AD | ○ |  |  | |
| F00.0 | Dementia in Alzheimer's disease with early onset | AD | ○ | ○ | ○ | |
| F00.1 | Dementia in Alzheimer's disease with late onset | AD | ○ | ○ | ○ | |
| F00.2 | Dementia in Alzheimer's disease, atypical or mixed type | AD | ○ | ○ | ○ | |
| F00.9 | Dementia in Alzheimer's disease, unspecified | AD | ○ | ○ | ○ | |
| G30 | Alzheimer's disease | AD | ○ |  |  | |
| G30.0 | Alzheimer's disease with early onset | AD | ○ | ○ |  | |
| G30.1 | Alzheimer's disease with late onset | AD | ○ | ○ |  | |
| G30.8 | Other Alzheimer's disease | AD | ○ | ○ |  | |
| G30.9 | Alzheimer's disease unspecified | AD | ○ | ○ |  | |
| F01 | Vascular dementia | VD | ○ |  |  | |
| F01.0 | Vascular dementia of acute onset | VD | ○ | ○ | ○ | |
| F01.1 | Multi-infarct dementia | VD | ○ | ○ | ○ | |
| F01.2 | Subcortical vascular dementia | VD | ○ | ○ | ○ | |
| F01.3 | Mixed cortical and subcortical vascular dementia | VD | ○ |  |  | |
| F01.8 | Other vascular dementia | VD | ○ |  |  | |
| F01.9 | Vascular dementia, unspecified | VD | ○ | ○ | ○ | |
| I67.3 | Binswanger's disease | VD | ○ |  |  | |
| F02.0 | Dementia in Picks disease | FTD | ○ | ○ | ○ | |
| G31.0 | Circumscribed brain atrophy | FTD | ○ | ○ |  | |
| A81.0 | Sporadic Creutzfeldt-Jakob disease | O | ○ |  |  | |
| F02.1 | Dementia in Creutzfeldt-Jacob disease | O | ○ |  |  | |
| F02.2 | Dementia in Huntington's disease | O | ○ | ○ | ○ | |
| F02.3 | Dementia in Parkinson's disease | O | ○ | ○ | ○ | |
| F02.4 | Dementia in HIV disease | O | ○ | ○ | ○ | |
| F10.6 | Mental and behavioral disorders due to use of alcohol – amnesic syndrome | O | ○ |  |  | |
| F02 | Dementia in other diseases classified elsewhere | N | ○ |  |  | |
| F02.8 | Dementia in other specified diseases classified elsewhere | N | ○ | ○ | ○ | |
| F03 | Unspecified dementia | N | ○ | ○ | ○ | |
| F05.1 | Delirium superimposed on dementia | N | ○ | ○ | ○ | |
| G31.1 | Senile degeneration of the brain | N | ○ |  |  | |
| G31.8 | Other specified degenerative diseases of the nervous system | N | ○ | ○ |  | |
| ICD10 – International Classification of Diseases-10, UKB – UK Biobank, JNHI – Japanese National Health Insurance, LSMC – Later-Stage Medical Care, AD – Alzheimer's disease, VD – vascular dementia, FTD – frontotemporal dementia, O – other dementia subtype, N – no subtype specified.  * Wilkinson T, Schnier C, Bush K, Rannikmae K, Henshall DE, Lerpiniere C, et al. Identifying dementia outcomes in UK Biobank: a validation study of primary care, hospital admissions and mortality data. Eur J Epidemiol. 2019 Jun;34(6):557-65. PMID: 30806901. doi: 10.1007/s10654-019-00499-1. | | | | | |  |
